# The Association of HLA-Class I and Class II with Hodgkin's Lymphoma in Iranian Patients

**DOI:** 10.1155/2014/231236

**Published:** 2014-05-21

**Authors:** Arezou Sayad, Mohammad Taghi Akbari, Mahshid Mehdizadeh, Abolfazl Movafagh, Abbas Hajifathali

**Affiliations:** ^1^Department of Medical Genetics, Shahid Beheshti University of Medical Sciences, Tehran 1985717443, Iran; ^2^Department of Medical Genetics, Faculty of Medical Science, Tarbiat Modares University, Tehran, Iran; ^3^Taleghani Bone Marrow Transplantation Center, Shahid Beheshti University of Medical Sciences, Tehran 1985717443, Iran; ^4^Pediatric Congenital Hematologic Disorders Research Center, Shahid Beheshti University of Medical Sciences, Tehran 1985717443, Iran

## Abstract

The Hodgkin's lymphoma disease (HD) is a common malignant neoplasm with germinal centre B-cell origin. It has been suggested that the HLA class I and class II regions have susceptibility effects on HD. In different ethnic groups, different HLA class I and class II alleles affect HD. As a result, there is no consensus which of the different HLA alleles confers susceptibility to HD. In this study, we aimed to ascertain the role of HLA class I and class II alleles in association with Hodgkin's lymphoma in Iranian patients. We performed a case-control genotyping study in 85 Iranian HD patients which were selected from the Bone Marrow Transplantation Department of Taleghani Hospital and 150 controls using the SSP-PCR. Our results demonstrated that the HLA-A*68, HLA-B*51, and HLA-DRB1*15 alleles were significantly more frequent in HD patients in comparison to controls (*P* = 0.026; OR = 6.188, *P* = 0.00008; OR = 2.86, *P* = 0.00006; OR = 5.315, resp.) and they have significant susceptibility effects on HD in Iranian population. There are reports of other populations with regard to consistency and inconsistency to our results. Further studies with large sample size or the meta-analysis are needed to explain the exact associations of HLA gene with HD.

## 1. Introduction

The Hodgkin's lymphoma disease (HD) is a common malignant neoplasm of germinal centre B-cell origin which is histopathologically characterized by the existence of very large Hodgkin Reed-Sternberg cell (HRS) [1]. The annual incidence of HD is about 2-3/100000 people, and this disease accounts for about 1% of all cancers worldwide [2]. HD is more frequent in two age groups, the first being in age 15–35 and the second being in over 55 years old [2]. Epstein Barr virus (EBV) is a susceptibility factor to HD. The precise mechanism remains unknown [3]. HLA genes play a crucial role in the control of immune response [4–7]. It is believed that HLA region can influence susceptibility to or protection against HD. Association of HD with HLA region was the first disease to be studied. For the first time, Amiel described association of HLA genes with HD [8]. Chakravarti et al. by studying HLA haplotype sharing between affected relatives demonstrated that around 60% of the cases in multiplex families were due to an HLA-linked susceptibility gene. According to these data, a recessive mode of inheritance for susceptibility to HD was suggested [9]. The HLA genotyping in familial Hodgkin's disease strongly supported the hypothesis of the susceptibility effect of the HLA genes on HD [10]. Some studies on the HLA class I and II regions and HD have proved positive association [11–16]. Studies to date have demonstrated specific alleles of HLA in correlation with HD. However, the different profiles of HLA alleles and haplotypes in HD patients have been studied in western populations and there are a few data in this regard in the eastern populations. Previously, we studied some genes, especially HLA genes, in Iranian patients with autoimmune disease [17–21]. In another population with different ethnic background, a different picture is expected. In the present study, for the first time, the associations of HLA class I and class II with HD in Iranian patients were investigated.

## 2. Materials and Methods

### 2.1. Patients and Controls

Eighty-five Iranian patients (mean age of 32 ± 2.7 years, range of 14–49 years) with HD from bone marrow transplantation department of Taleghani Hospital were selected. The diagnosis of HD was made by oncologist. Besides, one hundred fifty ethnically, age and sex matched healthy individuals (mean age of 33 years, range of 15–53 years) without personal or familial backgrounds of cancer or autoimmune disorders were selected from the same geographical area of the patients, as a control group. The subjects gave informed consent to participate. This study was approved by a local ethical committee.

### 2.2. DNA Extraction and HLA Genotyping

Genomic DNA from peripheral blood samples was isolated by applying salting out method. The HLA typing was carried out at the Tehran Medical Genetics Laboratory. HLA-A, HLA-B, HLA-DRB1 genotyping was performed based on SSP-PCR by HLA-READY GENE ABDR Kit (Inno-Train Diagnostic GmbH, Germany) according to the manufacturer's recommendation. The electrophoresis using 2% agarose gel was applied to amplified PCR products.

### 2.3. Statistical Analysis

Comparisons between the various HLA-A, HLA-B, and HLA-DRB alleles of patients with HD and controls were made using the chi-square and Fisher's exact tests. All the analyses were done using SPSS version 18.0 for windows software. The *P* value was corrected with Bonferroni correction. In statistics, the Bonferroni correction is a method used to counteract the problem of multiple comparisons. It is considered the simplest and most conservative method to control the family wise error rate. The null hypothesis of our study is to test the proportions of alleles comparing all other alleles not different between the two groups. The *P* values required rejecting null hypothesis for HLA-A, HLA-B, and HLA-DRB1 were calculated.

## 3. Results

Distributions of sex and age of HD patients and controls are summarized in Table 1. The allele frequencies of HLA-A, HLA-B, and HLA-DRB1 in patient and control groups are shown in Table 2. Low resolution HLA typing revealed that the HD group has a significant increased frequency of HLA-A*68 allele compared to control individuals, significantly (*P* = 0.026; OR = 6.188) (Table 2). As outlined in Table 2, the HLA-B*51 and HLA-DRB1*15 alleles were significantly more frequent among HD patient group than controls (*P* = 0.00008; OR = 2.86, *P* = 0.00006; OR = 5.315, resp.) (Table 2). The HLA-A*03 and HLA-DRB1*07 had lower frequency in HD patients than controls, but they did not remain significant after correction (*P* = 0.546; OR = 0.568, *P* = 0.299; OR = 0.387, resp.) (Table 2).

## 4. Discussion

HD is one of the multifactorial and heterogeneous malignancies of the immune system. In 1997, Ferraris et al. calculated that around 4.5% of HD was familial HD [22]. In a short period of time after introducing the serological technique for HLA typing, many studies on the association of the HLA allele and susceptibility to or protection against different disease were carried out. In 1979, for the first time, Amiel studied the association between HLA genes and HD [8]. Further investigations to unravel correlations of different HLA loci with HD, in familial and nonfamilial HD, were followed. In the present study which is the first one in Iran, we here investigated the association of HLA class I and class II with HD.

Our study indicates that, the HLA-A*68, HLA-B*51, and HLA-DRB1*15 alleles showed significant positive associations with HD and have had susceptibility effects on the disease, whereas HLA-A*03 and HLA-DRB1*07 alleles were more frequent in controls than HD patients (but they did not remain significant after correction). Haplotype analysis could not be calculated due to small sample size. In comparison to other populations as summarized in Table 3, the results of a study on a population with European ancestry, in 2011, determined that the DRB1*07:01 allele was decreased in HD patients and had negative association with HD, whereas the DRB1*15:01 allele showed positive association to HD [23] which was similar to our results. In contrast to our research, an increased frequency of HLA Bl8 in HD had been found by Amiel in 1967, Svejgaard and Bjorkholm in 1975 [8, 24, 25]. Taylor et al. indicated that DRB1*15:01 (OR = 1.93) and DRB1*01:01 (OR = 0.38) alleles had significant positive and negative association with HD, respectively [26]. A study on Egyptian population also demonstrated that the HLA-DRB1*04:03 and *12:02 alleles had susceptibility effect on HD [27]. Klitz et al., in a case-control study on the Caucasians with nonfamilial HD, showed that the DRB1*15:01-DQA1*01:02-DQB1*06:02 haplotype had significant increased risk in one HD subtype (nodular sclerosis). Also they reported that DRB1*16:01-DQA1*01:02-DQB1*05:02, DRB1*04:04-DQA1*03:01-DQB1*03:02, DRB1*07:01-DQA1*02:01-DQB1*03:03, and DRB1*14:01-DQA1*01:01-DQB1*05:03 haplotypes were decreased in the HD patients and had protection effects on HD [11]. One study in 2002 among familial HD patients showed the significant association of DRB1*15:01-DQA1*01:02-DQB1*06:02 haplotype to the development of familial HD [28].

The above descriptions demonstrated that there were differences in susceptible or protective alleles of HLA genes between populations. These differences may be resulting from their ancestries or the small sample size. In this study, we showed susceptible alleles in Iranian HD patients. Meta-analysis of results among populations, study on large sample size (especially on antigen binding groove), and more and precise studies in this field are necessary to clear the causes exact susceptible or protective alleles of HLA gene in HD.

In conclusion, the HLA-A*68, HLA-B*51, and HLADRB1*15 alleles showed significant susceptibility effects on HD in Iranian population. However, there are also reports of other populations with regard to consistency and inconsistency to our results. Ethnic variations among different populations may explain these controversies. Further studies with large sample size are needed to explain exact associations of HLA gene with HD.

## Figures and Tables

**Table 1:** Distributions of sex and age of HD patients and control group.

Variables	HD patients	Controls
Female/Male (no. (%))	37/48 (43/57%)	59/91 (39/61%)
Age (mean ± SD, years)	32 ± 2.7	33 ± 3.1
Age range (years)	14–49	15–53

**Table 2:** The allele frequencies of HLA-A, HLA-B, HLA-DRB1 in HD patients and control groups.

Alleles	HD patients *n* = 170 (%)	Controls *n* = 300 (%)	*P* value^a^	PC value^b^	OR (95% CI)^c^
HLA-A*01	15 (9%)	30 (10%)	0.67	>0.999	0.871 (0.361–2.098)
HLA-A*02	36 (21%)	63 (21%)	0.96	>0.999	1.011 (0.542–1.885)
HLA-A*03	20 (12%)	57 (19%)	0.04	0.546	0.568 (0.27–1.192)
HLA-A*11	17 (10%)	27 (9%)	0.72	>0.999	1.12 (0.472–2.654)
HLA-A*23	5 (3%)	9 (3%)	0.97	>0.999	0.98 (0.218–4.394)
HLA-A*24	36 (21%)	51 (17%)	0.26	>0.999	1.31 (0.688–2.491)
HLA-A*26	3 (2%)	15 (5%)	0.07	>0.999	0.341 (0.062–1.858)
HLA-A*29	5 (3%)	9 (3%)	0.97	>0.999	0.98 (0.218–4.394)
HLA-A*30	7 (4%)	12 (4%)	0.95	>0.999	1.03 (0.284–3.729)
HLA-A*31	5 (3%)	12 (4%)	0.55	>0.999	0.727 (0.173–3.050)
HLA-A*32	5 (3%)	6 (2%)	0.51	>0.999	1.48 (0.291–7.517)
HLA-A*33	6 (4%)	6 (2%)	0.31	>0.999	1.79 (0.379–8.447)
HLA-A*68	10 (6%)	3 (1%)	0.002	**0.026**	**6.188 (1.059–36.053)**
HLA-B*07	7 (4%)	15 (5%)	0.66	>0.999	0.816 (0.197–3.37)
HLA-B*08	2 (1%)	12 (4%)	0.08	>0.999	0.286 (0.027–2.947)
HLA-B*13	2 (1%)	9 (3%)	0.20	>0.999	0.38 (0.034–4.133)
HLA-B*14	2 (1%)	9 (3%)	0.20	>0.999	0.38 (0.034–4.133)
HLA-B*15	7 (4%)	9 (3%)	0.52	>0.999	1.38 (0.291–6.537)
HLA-B*18	5 (3%)	12 (4%)	0.55	>0.999	0.72 (0.139–3.71)
HLA-B*27	5 (3%)	8 (3%)	0.86	>0.999	1.1 (0.190–6.345)
HLA-B*35	34 (20%)	53 (18%)	0.53	>0.999	1.16 (0.553–2.431)
HLA-B*38	10 (6%)	16 (5%)	0.80	>0.999	1.1 (0.312–3.868)
HLA-B*39	7 (4%)	9 (3%)	0.52	>0.999	1.38 (0.291–6.537)
HLA-B*40	2 (1%)	6 (2%)	0.50	>0.999	0.583 (0.048–7.04)
HLA-B*41	2 (1%)	9 (3%)	0.20	>0.999	0.385 (0.035–4.188)
HLA-B*44	10 (6%)	28 (9%)	0.18	>0.999	0.607 (0.190–1.929)
HLA-B*48	2 (1%)	6 (2%)	0.50	>0.999	0.583 (0.048–7.04)
HLA-B*49	3 (2%)	12 (4%)	0.18	>0.999	0.431 (0.059–3.115)
HLA-B*50	3 (2%)	15 (5%)	0.07	>0.999	0.34 (0.048–2.363)
HLA-B*51	54 (32%)	42 (14%)	0.000003	**0.00008**	**2.86 (1.406–5.814)**
HLA-B*52	5 (3%)	12 (4%)	0.55	>0.999	0.72 (0.139–3.71)
HLA-B*54	2 (1%)	0 (0%)	0.25	>0.999	2.78 (0.022–337.713)
HLA-B*55	2 (1%)	6 (2%)	0.50	>0.999	0.583 (0.048–7.04)
HLA-B*57	2 (1%)	3 (1%)	0.85	>0.999	1.17 (0.072–18.886)
HLA-B*58	2 (1%)	9 (3%)	0.20	>0.999	0.38 (0.034–4.133)
HLA-DRB1*01	8 (5%)	12 (4%)	0.71	>0.999	1.18 (0.339–4.099)
HLA-DRB1*03	12 (7%)	30 (10%)	0.28	>0.999	0.68 (0.261–1.768)
HLA-DRB1*04	9 (5%)	15 (5%)	0.88	>0.999	1.06 (0.333–3.369)
HLA-DRB1*07	7 (4%)	30 (10%)	0.02	0.299	0.387 (0.122–1.21)
HLA-DRB1*08	0 (0%)	3 (1%)	0.99	>0.999	0.58 (0.0001–2918.205)
HLA-DRB1*09	2 (1%)	3 (1%)	0.99	>0.999	1.17 (0.102–13.374)
HLA-DRB1*10	10 (6%)	15 (5%)	0.68	>0.999	1.18 (0.384–3.624)
HLA-DRB1*11	70 (41%)	120 (40%)	0.80	>0.999	1.05 (0.571–1.928)
HLA-DRB1*12	3 (2%)	0 (0%)	0.13	>0.999	5.38 (0.001–27068.86)
HLA-DRB1*13	12 (7%)	30 (10%)	0.28	>0.999	0.68 (0.261–1.768)
HLA-DRB1*14	10 (6%)	21 (7%)	0.63	>0.999	0.83 (0.287–2.4)
HLA-DRB1*15	24 (14%)	9 (3%)	0.000004	**0.00006**	**5.315 (1.806–15.637)**
HLA-DRB1*16	3 (2%)	12 (4%)	0.18	>0.999	0.431 (0.075–2.444)

The significant *P* value after Bonferroni correction and OR are shown in bold. ^a^Value of chi-square and Fisher's exact test without correction. ^b^Adjusted Bonferroni *P* value. ^c^corrected 95% confidence interval for odds ratio.

*n*: number of alleles, NS: not significant, and HD: Hodgkin's lymphoma.

**Table 3:** The susceptible and protective alleles or haplotypes in HD.

Susceptible and protective alleles or haplotypes	Study
Susceptible allele: HLA-B18	Amiel 1967, Svejgaard et al., 1975, Bjorkholm et al., 1975, [8, 24, 25]

Susceptible haplotype: DRB1*15:01-DQA1*01:02-DQB1*06:02 Protective haplotypes: DRB1*16:01-DQA1*01:02-DQB1*05:02, DRB1*04:04-DQA1*03:01-DQB1*03:02, DRB1*07:01-DQA1*02:01-DQB1*03:03 and DRB1*14:01-DQA1*01:01-DQB1*05:03	Klitz et al., 1994, [11]

Susceptible allele: HLA-DRB1*15:01 Protective allele: HLA-DRB1*01:01	Taylor et al., 1996, [26]

Susceptible haplotype: DRB1*15:01-DQA1*01:02-DQB1*06:02	Harty et al., 2002, [28]

Susceptible alleles: HLA-DRB1*04:03 and HLA-DRB1*12:02	Al-Tonbary et al., 2004 [27]

Susceptible allele: HLA-DRB1*15:01 Protective allele: HLA-DRB1*07:01	Moutsianas et al., 2011, [23]

Susceptible alleles: HLA-A*68, HLA-B*51 and HLA-DRB1*15	Present study

## References

[1] Hodgkin T (1832). On some morbid experiences of the absorbent glands and spleen. *Medico-Chirurgical Transactions*.

[2] Horning SJ, Lichtman MA, Beutler E, Kipps TJ (2006). Hodgkin lymphoma. *Williams Hematology*.

[3] Depil S, Moralès O, Auriault C (2004). Hodgkin’s disease and Epstein-Barr virus. *Annales de Biologie Clinique*.

[4] Brown JH, Jardetzky TS, Gorga JC (1993). Three-dimensional structure of the human class II histocompatibility antigen HLA-DR1. *Nature*.

[5] Germain RN (1994). MHC-dependent antigen processing and peptide presentation: providing ligands for T lymphocyte activation. *Cell*.

[6] Hammer J (1995). New methods to predict MHC-binding sequences within protein antigens. *Current Opinion in Immunology*.

[7] Hammer J, Gallazzi F, Bono E (1995). Peptide binding specificity of HLA-DR4 molecules: correlation with rheumatoid arthritis association. *Journal of Experimental Medicine*.

[8] Amiel J, Teraski PI (1967). Study of the leukocyte phenotypes in Hodgkin’s disease. *Histocompatibility Testing*.

[9] Chakravarti A, Halloran SL, Bale SJ, Tucker MA (1986). Etiological heterogeneity in Hodgkin’s disease: HLA linked and unlinked determinants of susceptibility independent of histological concordance. *Genetic Epidemiology*.

[10] Hors J, Steinberg G, Andrieu JM (1980). HLA genotypes in familial Hodgkin’s disease. Excess of HLA identical affected sibs. *European Journal of Cancer and Clinical Oncology*.

[11] Klitz W, Aldrich CL, Fildes N, Horning SJ, Begovich AB (1994). Localization of predisposition to Hodgkin disease in the HLA class II region. *American Journal of Human Genetics*.

[12] Oza AM, Tonks S, Lim J, Fleetwood MA, Lister TA, Bodmer JG (1994). A clinical and epidemiological study of human leukocyte antigen-DPB alleles in Hodgkin’s disease. *Cancer Research*.

[13] Tonks S, Oza AM, Lister TA, Bodmer JG (1992). Association of HLA-DPB with Hodgkin’s disease. *The Lancet*.

[14] Marshall WH, Barnard JM, Buehler SK, Crumley J, Larsen B (1977). HLA in familial Hodgkin’s disease. Results and a new hypothesis. *International Journal of Cancer*.

[15] Niens M, van den Berg A, Diepstra A (2006). The human leukocyte antigen class I region is associated with EBV-positive Hodgkin’s lymphoma: HLA-A and HLA complex group 9 are putative candidate genes. *Cancer Epidemiology Biomarkers and Prevention*.

[16] Niens M, Jarrett RF, Hepkema B (2007). HLA-A*02 is associated with a reduced risk and HLA-A*01 with an increased risk of developing EBV+ Hodgkin lymphoma. *Blood*.

[17] Sayad A, Allameh A, Sayad A (2013). The association of -475 and -631 interleukin-2 gene polymorphism with multiple sclerosis in Iranian patients. *Cell Journal*.

[18] Sayad A, Allameh A, Sayad A, Norouzinia M, Sarzaeem A (2013). The influence of -330 IL-2 gene polymorphism on relapsing remitting and secondary progressive course multiple sclerosis in Iranian patients. *Neurology Asia*.

[19] Sayad A, Akbari MT, Pajouhi M, Mostafavi F, Kazemnejad A, Zamani M (2013). The role of the gender on the HLA-DRB1 and DQB1 association with type 1 diabetes mellitus in Iranian patients. *Cell Journal*.

[20] Sayad A, Akbari MT, Pajouhi M, Mostafavi F, Zamani M (2012). The influence of the HLA-DRB, DQB and polymorphic positions of the HLA-DR*β*1 and HLA-DQ*β*1 molecules on risk of Iranian type 1 diabetes mellitus patients. *International Journal of Immunogenetics*.

[21] Hajifathali A, Sayad A, Sayad A (2012). The association of -308 TNF*α* polymorphism and multiple sclerosis in Iranian patients. *Journal of Biology and Today's World*.

[22] Ferraris AM, Racchi O, Rapezzi D, Gaetani GF, Boffetta P (1997). Familial Hodgkin’s disease: a disease of young adulthood?. *Annals of Hematology*.

[23] Moutsianas L, Enciso-Mora V, Ma YP (2011). Multiple Hodgkin lymphoma-associated loci within the HLA region at chromosome 6p21.3. *Blood*.

[24] Svejgaard A, Platz P, Ryder LP (1975). HL A and disease associations. A survey. *Transplantation Reviews*.

[25] Bjorkholm M, Holm G, Johansson B, Mellstedt H, Moller E (1975). A prospective study of HL A antigen phenotypes and lymphocyte abnormalities in Hodgkin’s disease. *Tissue Antigens*.

[26] Taylor GM, Gokhale DA, Crowther D (1996). Increased frequency of HLA-DPB1*0301 in Hodgkin’s disease suggests that susceptibility is HVR-sequence and subtype-associated. *Leukemia*.

[27] Al-Tonbary Y, Abdel-Razek N, Zaghloul H, Metwaly S, El-Deek B, El-Shawaf R (2004). HLA class II polymorphism in Egyptian children with lymphomas. *Hematology*.

[28] Harty LC, Lin AY, Goldstein AM (2002). HLA-DR, HLA-DQ, and TAP genes in familial Hodgkin disease. *Blood*.

